# Vacuum Rabi splitting in a plasmonic cavity at the single quantum emitter limit

**DOI:** 10.1038/ncomms11823

**Published:** 2016-06-13

**Authors:** Kotni Santhosh, Ora Bitton, Lev Chuntonov, Gilad Haran

**Affiliations:** 1 Department of Chemical Physics, Weizmann Institute of Science, POB 26, Rehovot 76100, Israel; 2 Department of Chemical Research Support, Weizmann Institute of Science, Rehovot 76100, Israel; 3 Schulich Faculty of Chemistry, Technion-Israel Institute of Technology, Haifa 32000, Israel

## Abstract

The strong interaction of individual quantum emitters with resonant cavities is of fundamental interest for understanding light–matter interactions. Plasmonic cavities hold the promise of attaining the strong coupling regime even under ambient conditions and within subdiffraction volumes. Recent experiments revealed strong coupling between individual plasmonic structures and multiple organic molecules; however, strong coupling at the limit of a single quantum emitter has not been reported so far. Here we demonstrate vacuum Rabi splitting, a manifestation of strong coupling, using silver bowtie plasmonic cavities loaded with semiconductor quantum dots (QDs). A transparency dip is observed in the scattering spectra of individual bowties with one to a few QDs, which are directly counted in their gaps. A coupling rate as high as 120 meV is registered even with a single QD, placing the bowtie-QD constructs close to the strong coupling regime. These observations are verified by polarization-dependent experiments and validated by electromagnetic calculations.

## Abstract (web summary)

Strong coupling at the limit of a single quantum emitter has not been reported. Here, Santhosh *et al.* show a transparency dip is observed in the scattering spectra of individual silver bowties with one to a few quantum dots, placing the plasmonic bowtie-quantum dot constructs close to the strong coupling regime.

## Graphical abstract

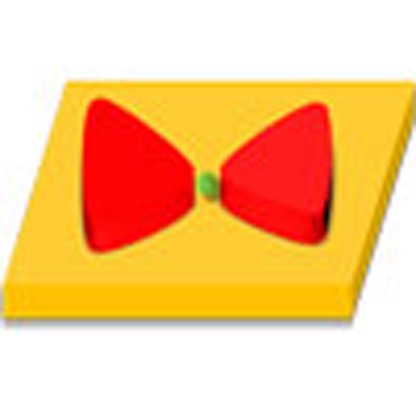

The interaction of emitters with an optical cavity falls within the realm of cavity quantum electrodynamics^1^. Approaching the limit of strong coupling between individual quantum emitters and resonant cavities is important for multiple advanced optical applications, such as quantum information processing^2^,^3^ and quantum communication^4^,^5^. Strong coupling can be observed through the phenomenon of vacuum Rabi splitting in the optical spectra of the joint systems, as well as in the appearance of non-classical photon correlations. The strength of the interaction depends on the ratio of the quality factor of the cavity to the mode volume, *Q/V*. In optical cavities made of, for example, photonic crystals^6^ or micropillars^7^, the fundamental laws of diffraction set a strict limitation on the cavity size, which cannot be smaller than half the wavelength of the interacting photon. This in turn limits how small *V* can be and mandates a very high *Q*, to attain the strong coupling regime. Obtaining such a high-quality factor requires demanding experimental conditions such as cryogenic temperatures and ultra-narrow frequency light sources.

Cavities made of materials that can sustain surface plasmon (SP) excitations can beat the diffraction limit by focusing intense electromagnetic fields into volumes much smaller than the wavelength of light^8^. Such cavities should simplify considerably the experimental conditions required for strong light–matter interactions and may allow quantum optical experiments to be conducted under ambient conditions.

Noble metals are most often used for generating structures sustaining SPs at visible wavelengths. Although the SP relaxation times in such structures are ultrafast, severely limiting *Q*, their mode volume is drastically reduced compared with photonic cavities. In recent years, there has been much interest in studies of coupling between SPs, either propagating or localized, and quantum emitters such as molecules^9^. These experiments involved multiple molecules and often multiple or extended plasmonic structures as well. In recent times, strong coupling has also been probed at the level of a single plasmonic device. Thus, Halas and colleagues^10^ used gold dimers and molecular J-aggregates to observe a Rabi splitting of ∼230 meV. Shegai and colleagues^11^ observed strong coupling using either a silver nanorod or a silver nanoprism^12^, in both cases employing J-aggregates as the quantum emitters. In all of these experiments, the interaction involved hundreds of quantum emitters or more. However, for quantum information operations one needs to approach the limit of a single quantum emitter coupled to the cavity.

In this study, we show that one can indeed observe strong coupling in the limit of a single quantum emitter. In particular, we use silver bowtie plasmonic cavities and couple them to semiconductor quantum dots (QDs). Scattering spectra registered from individual plasmonic cavities containing one to a few QDs show vacuum Rabi splitting, indicating that the strong coupling regime is approached in these systems. Polarization-dependent experiments verify that the observed Rabi splitting is due to the coupling of the longitudinal plasmon resonance of the bowties with the QDs.

## Results

### Construction of plasmonic cavities with QDs

Inspired by recent computational studies, suggesting that strong coupling with individual emitters can be achieved in the gaps of plasmonic structures^13^, we turned to silver bowties, which can be fabricated with a small gap^14^. Silver is preferred as a material for plasmonic devices due to its relatively low SP damping and hence the high achievable quality factors of such devices compared with other metals^15^. By varying the structural parameters of the bowties, including side length and gap size, the localized SP excitations of bowties manufactured by electron-beam lithography can be tuned to resonance with the quantum emitter of choice, while maintaining gaps of the order of ∼20 nm. To achieve strong coupling, the quantum emitter should possess a large oscillator strength and a narrow linewidth. QDs have several advantages over organic emitters in relation to the aforementioned characteristics^16^ and (most importantly) are also significantly more photostable. They can also be observed by electron microscopy and therefore can be directly counted. Commercially available CdSe/ZnS QDs were employed in the present study. Optical spectroscopy and electron microscopy studies showed that the sample is mildly heterogeneous and the diameter of the QD varies between 6 and 8 nm.

**Figure 1:**
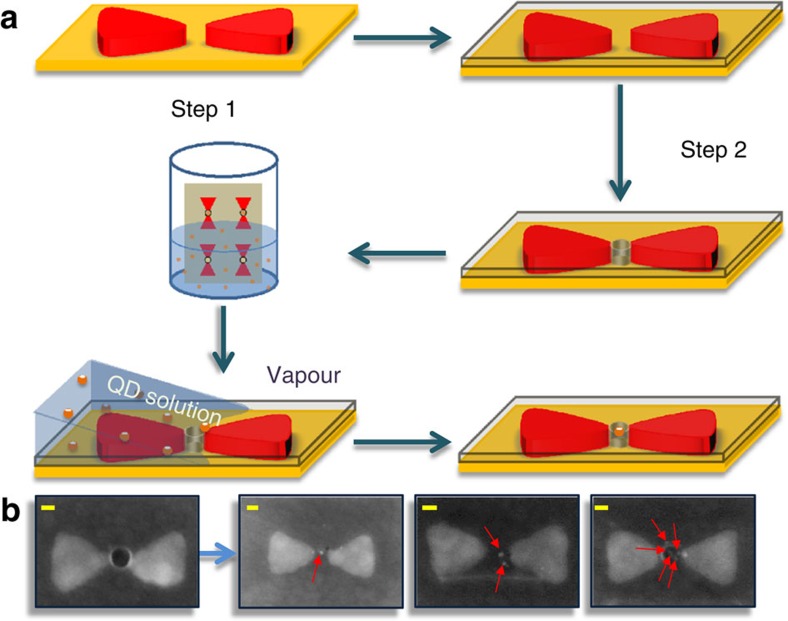
Construction of bowties with quantum dots in their gaps. (**a**) Schematic illustration of the two-step lithography process for making holes at the centre of bowtie structures and the interfacial capillary force assisted method for driving QDs into the holes. (**b**) Scanning electron microscope images of bowties with one, two and multiple QDs in their gaps (from left to right). The positions of the QDs are marked by red arrows. Scale bars, 20 nm (yellow).

To position QDs within the gaps of bowties, we made use of interfacial capillary forces^17^ to drive the QDs into lithographically patterned holes in the bowtie gaps. This simple method avoids additional steps such as chemical modification of the QD/nanostructure surfaces. The process, described in Fig. 1 and in the Methods section, led to the trapping in bowtie gaps of a number of QDs that varied from one to several, as could be ascertained by scanning electron microscopy.

### Spectroscopy demonstrates Rabi splitting

Dark-field microspectroscopy was used to characterize the plasmonic behaviour of every single bowtie by measuring its scattering spectrum. As shown in Supplementary Fig. 1, empty bowties possess two modes, a transverse mode at ≈2.05 eV and a longitudinal mode at ≈1.9 eV, the latter being due to dipolar coupling of the two parts of each bowtie. The absorption and emission spectra of the QDs are shown in Supplementary Fig. 2 and demonstrate that the optical transitions of the QDs are in resonance with the plasmon excitations of the bowties. (See also Supplementary Note 1 for further discussion on the role of the absorption and emission spectra in coupling to the plasmonic cavity.)

**Figure 2:**
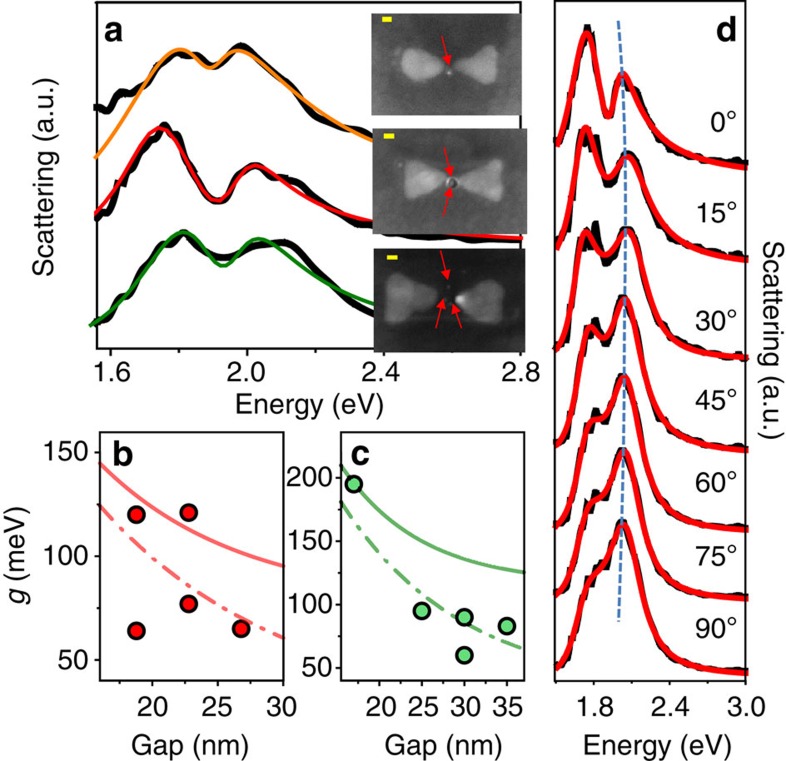
Strong coupling of plasmons and quantum emitters. (**a**) Scattering spectra of bowties with (from top to bottom) one, two and three QDs in the gap, respectively. All spectra show a transparency dip due to Rabi splitting. The black lines are experimental data and the coloured lines are fits to the coupled oscillator model. Insets show the scanning electron microscope images of the bowties. The positions of the QDs are marked by red arrows. Scale bars, 20 nm (yellow). (**b**,**c**) Coupling rates as a function of gap size for bowties with one QD ((**b**) red symbols) and two QDs ((**c**) green symbols). The errors in the coupling rate values, obtained from the fitted functions, are estimated to be 2–5 meV. The continuous lines represent the numerically calculated coupling rates at two configurations along the centre line of the bowtie: with the QDs almost touching one of the prisms (continuous lines) or with the QDs at the centre of the bowtie (dashed-dotted lines). In the experiments, the QDs may be positioned away from the centre line so that their coupling rates are lower than the calculated lines. (**d**) Polarization series of the middle bowtie structure in **a**. As the polarization of the excitation light is rotated from the direction parallel to the bowtie long axis to perpendicular to it, the transparency dip in the spectrum gradually vanishes, indicating that it indeed originates in the coupling of the QD exciton with the longitudinal plasmon resonance of the bowtie.

Figure 2a shows bowties with QDs in their gaps whose scattering spectra present transparency dips indicative of Rabi splitting. Overall, we collected scattering spectra from 21 QD-containing bowties, out of which 14 spectra showed a definite signature of splitting. Thus, the top bowtie in Fig. 2a, with a single QD and a particularly small gap (∼19 nm), presents a clear Rabi splitting feature. Similarly, Rabi splitting is observed in the spectra of the two other bowties shown in Fig. 2a, with two and three QDs and gaps of 17 and 30 nm, respectively. The spectra in this figure, as well as other spectra showing Rabi splitting, were fitted using the coupled oscillator model^18^ (see Methods). This model represents the plasmon mode and the exciton of the quantum emitter as coupled harmonic oscillators exchanging energy reversibly. The coupling rate characterizing the interaction is given by *g*=**μ**·**E**, where **μ** is the transition dipole moment of the exciton and **E** is the electric field of the plasmon. This coupling leads to the generation of upper (*Ω*_+_) and lower (*Ω*_−_) plasmon-exciton hybrid states, with a transparency dip in between^13^,^19^. The Rabi splitting values, *Ω*_+_−*Ω*_−_, can be obtained directly from the fitted curves, and for the three examples in Fig. 2a they are 176, 288 and 224 meV, respectively. These values are comparable to recently reported values of Rabi splittings of J-aggregates with individual plasmonic structures^10^,^12^, although hundreds of quantum emitters were involved in the latter. Not all bowties loaded with a single QD showed a clear transparency dip in the scattering spectrum. Instead, Rabi splitting manifested itself in a significant spectral broadening as compared with that of an empty bowtie (Supplementary Fig. 3), indicating coupling between plasmons and excitons. Splitting was readily observed in bowties with several QDs; see Supplementary Fig. 4 for additional spectra.

Coupling rates obtained from the fits of data sets with one and two QDs are plotted as a function of gap size in Fig. 2b,c. The figures also contain theoretical estimates of the coupling rates, based on the numerical calculations described below. The coupling rate depends strongly on the gap size of each bowtie. For instance, for two QDs the coupling rate is more than doubled when the gap size changes from 25 to 17 nm. In general, the coupling increases with the number of quantum emitters (*N*) (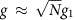, *g*_1_ being the coupling rate for one quantum emitter^1^), although in our experiments this dependence is partially masked by the dependence on gap size and QD position variations. Although the values of the coupling rates obtained from the fits are smaller than the values of the plasmon widths (which are ∼400 meV), significant splittings are observed, indicating that the strong coupling regime has indeed been attained, or is close^9^. Supplementary Fig. 5 shows histograms of the values of all parameters obtained from the coupled oscillator fits to measured spectra.

To obtain an unequivocal proof that the splitting in the spectra is due to strong (or close-to-strong) coupling of the bowtie longitudinal plasmon mode and the quantum emitter's exciton, we resorted to polarization-dependent experiments in which the polarization of the excitation source was rotated. Figure 2d shows an example of a polarization series measured on the bowtie with two QDs from Fig. 2a. When the excitation is polarized along the longitudinal direction of the bowtie (that is, along its long axis), the spectrum shows two peaks due to Rabi splitting. As the polarization is rotated to the transverse direction, the double-peaked spectrum is gradually replaced with the single-peaked spectrum of the transverse mode. Similar results with other bowties are shown in Supplementary Fig. 6. These results clearly indicate that the transparency dips in the scattering spectra are due to a genuine coupling of the plasmon and QD excitations.

## Discussion

**Figure 3:**
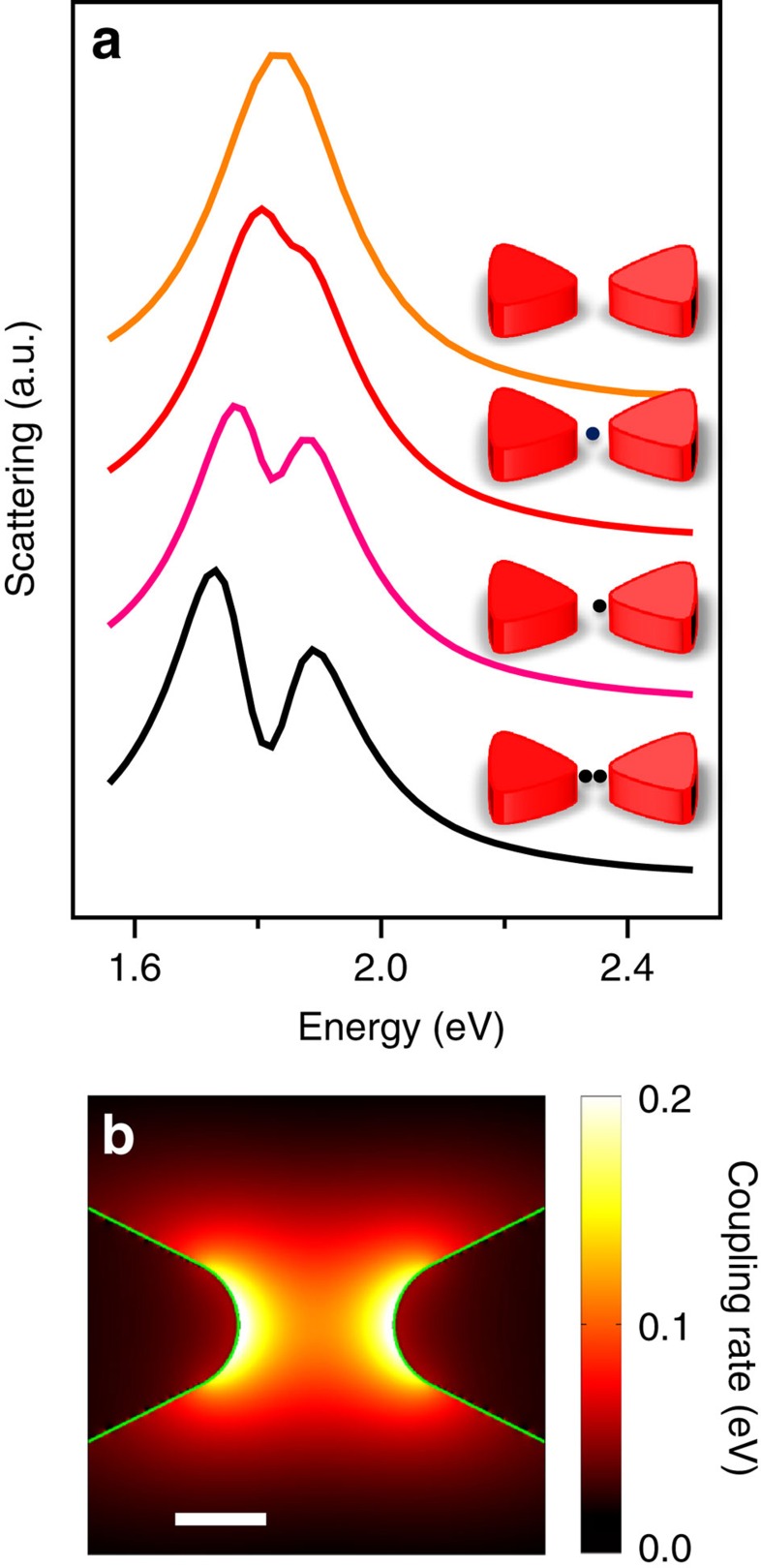
Electromagnetic simulations of strongly coupled plasmonic structures. (**a**) Simulated scattering spectra of bowties with one and two QDs organized as indicated in the inset structures. (**b**) Distribution of the coupling rate (in eV) of a quantum emitter with an oscillator strength of 0.6 in a bowtie structure. The white bar represents 10 nm.

The experimental results described above show that the positioning of one or a few QDs within a silver bowtie can bring this plasmonic cavity very close to the strong coupling regime. To support the experimental results and shed further light on them, we performed electromagnetic calculations, using the boundary element method^20^. The simulations revealed some interesting facts. The top spectrum in Fig. 3a is the calculated scattering spectrum of an empty bowtie. The spectrum for a bowtie with a single QD at the centre of the gap is shown below, demonstrating a small change compared with the empty bowtie. However, when the QD is positioned closer to one of the prisms constituting the bowtie, or the number of QDs in the hotspot is increased from one to two, a more significant transparency dip appears. The Rabi splitting is observed in both calculated scattering and extinction spectra (the latter not shown). Importantly, no such splitting is found when the simulation is repeated with a single metallic prism rather than a bowtie structure.

The simulation allows us to directly calculate the coupling rate for a quantum emitter with an oscillator strength similar to a QD (∼0.6)^21^,^22^,^23^. The spatial distribution of the coupling rate across the gap is shown in Fig. 3b, clearly indicating that the electromagnetic field in the hotspot is not uniform and is concentrated at the edges rather than at the centre. Indeed, in many of the bowties exhibiting strong coupling (Fig. 2 and Supplementary Figs 3 and 4) the QDs are close to one of the edges, in agreement with the simulation results. We estimate from the simulation that at the centre of a bowtie structure *g* is ∼100 meV, whereas *g* is as high as 200 meV very close to one of the prisms.

In conclusion, we have successfully integrated lithographically fabricated silver bowties with one to a few QDs that reside exactly within the plasmonic cavity. The very small plasmon mode volumes of the bowties allowed us to demonstrate vacuum Rabi splitting in the limit of an individual quantum emitter. A key advantage of using QDs is that we can directly count them under the electron microscope and verify the relation between coupling and the number of quantum emitters within each cavity. An important question to ask is: what value of the coupling may deem it ‘strong'? An often-used rule of thumb for the strong coupling regime is 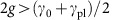, where *γ*_0_ and *γ*_pl_ are the QD and plasmonic cavity linewidths, respectively^9^,^24^. The maximal coupling rates obtained in this work for two and three QDs (∼200 meV; Fig. 2b,c and Supplementary Fig. 5d) fulfil the above criterion, whereas the maximum value obtained for a single QD (120 meV) is somewhat smaller. This shows that the constructs of QDs with plasmonic cavities studied here are definitely close to the strong coupling regime. In future experiments we plan to significantly increase the coupling between individual QDs and bowties. To this end, we will reduce the mode volume of the bowties, which can be achieved by decreasing their gap size and improving control over their shape in the fabrication process. Further improvement in QD positioning methodology will allow us to locate a single QD reproducibly at the position of the highest field within a bowtie cavity. Such improvements will enable systematic variation of experimental parameters and therefore provide additional proofs for strong coupling. Importantly, our results also pave the way to more sophisticated quantum nonlinear optics experiments^25^. In particular, by probing time-resolved photoluminescence of the coupled QD-bowtie system we hope to directly demonstrate quantum correlations^2^,^26^.

## Methods

### Materials

Glass/ITO (10 × 10 × 1.1 mm) substrates were purchased from Xin Yan Technology Ltd., China. The ITO layer is 180 nm thick and is characterized by 83% transparency to visible light and a sheet resistance of 10 Ω^−2^. Poly(methyl methacrylate) 950 K A2 (PMMA) electron beam resist, used for the fabrication of bowties, was procured from MicroChem. Chromium (used as an adhesive layer) and silver were obtained from Kurt J. Lesker. Water-soluble mercapto-undecanoic acid capped CdSe/ZnS core/shell nanocrystals (QDs) were acquired from MK Impex Corp.

### Electron beam lithography and fabrication of silver bowties

Before fabrication, glass/ITO substrates were cleaned with acetone followed by isopropanol for 3 min in each step. The cleaned glasses were dried using a nitrogen flow and then PMMA was spin-coated at a speed of 6,000 r.p.m. for 50 s to achieve a thickness of 60 nm, followed by baking at 180 °C for 90 s. The PMMA-coated glasses were loaded into a Raith E_line Plus electron beam lithography system and the PMMA was exposed to define the shape of bowties. The accelerating voltage used for the exposure was 30 kV and the beam current was 30 pA. The design consisted of matrices of bowties, with each matrix hosting 64 bowties. Each bowtie was separated by 10 μm from its neighbouring partner to guarantee no interaction. To remove the exposed PMMA, the substrates were developed using 1:3 methyl isobutyl ketone:isopropyl alcohol for 30 s, followed by immersion in a stopper (isopropyl alcohol) for 30 s and drying with a nitrogen flow.

An electron beam evaporator (Odem) was used for the metal deposition on the patterned substrates. Initially, chromium was evaporated to deposit a 2-nm adhesion layer, followed by silver. Different prism lengths from 75 to 200 nm and thicknesses from 20 to 35 nm were examined and finally the dimensions were fixed to a length of ≈85 nm and a thickness of ≈30 nm. These bowties showed a plasmon resonance that overlapped the QD exciton frequency. After metallization, a lift-off step was performed in acetone for 5 min to obtain silver bowties on the glass/ITO substrate.

A second stage of E-beam lithography was performed, to expose holes in the gap regions of the bowties. A 60-nm layer of PMMA was spin-coated on the substrate and then the gap regions of the bowties were exposed to create 10–30 nm holes, into which the QDs were driven. (See below and in Fig. 1 of the main text.) To reach an overlay accuracy as high as a few nanometres in the second exposure, we made use of alignment marks that were fabricated in the first E-beam exposure step.

### Incorporation of QDs into bowtie structures

To incorporate QDs into the bowtie gaps we adopted a method originally developed by Alivisatos and colleagues^17^. Substrates with bowties and holes in the PMMA layer prepared as described above were placed vertically in an aqueous solution of QDs. Controlled solvent evaporation was then used to exert a capillary force along the receding line of contact of the QD solution and drive QDs successfully into the holes. By varying the QD concentration in solution (from 10 to 100 nM), bowties with one to a few QDs in the gap were successfully obtained (Fig. 1). Although this method is limited in controlling the exact position of the QDs within the gap, it was found to be facile and relatively reproducible.

### Bulk absorption and emission measurements of QDs

Steady-state absorption and emission spectral measurements (Supplementary Fig. 2) were carried out on a Cary-100 ultraviolet–visible spectrophotometer (Varian) and a Fluorolog-3 spectrofluorimeter (Horiba Jobin Yvon), respectively. Extinction coefficients of the QDs were estimated by employing the method developed by Peng and colleagues^23^.

### Dark-field microspectroscopy

Our dark-field microspectrometer is described schematically in Supplementary Fig. 8. Briefly, scattering spectra of single bowties, either with or without QDs, were measured with an inverted microscope equipped with a dark-field condenser, a 75 W Xenon lamp (Olympus) and a × 100 oil-immersion objective with a numerical aperture of 0.6. A SpectraPro-150 spectrograph with a 1,200 g mm^−1^ grating (Acton) and a Newton spectroscopy charge-coupled device camera (Andor Technology) were used to disperse scattered photons and register spectra. Raw spectra were smoothed using a Fourier low-pass filter. Before the spectral measurements, the camera was calibrated in reference to the spectrum of fluorescent dyes. Polarization of the excitation light was controlled by a combination of a polarizer and a selecting sector, such that essentially only *s*-polarized light reached the sample.

### Data fitting to the coupled oscillator model

Scattering spectra were fitted to the following equation, which represents the scattering of two coupled harmonic oscillators at frequency *ω*^18^:


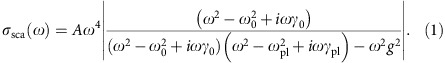


In this expression *ω*_pl_ and *ω*_0_ are the plasmon and QD resonance frequencies, respectively, whereas *γ*_pl_ and *γ*_0_ are the corresponding linewidths. *g* is the coupling rate and *A* is a scaling parameter. Parameters obtained from fits to all our data sets are shown in Supplementary Fig. 5. We fixed the value of *γ*_0_ to 130 meV, which is the measured linewidth of the fluorescence spectra of individual QDs (Supplementary Fig. 2). In the case of some of the data sets with a single QD in the gap, where broadening of the spectrum was observed, although no clear splitting, we also fixed the value of *γ*_pl_ to 385 meV, which is the average value of this parameter obtained from multiple spectra of empty bowties.

### Electromagnetic simulations

Numerical simulations of the silver bowties were performed using the boundary-element method as implemented in MNPBEM—a toolbox developed by Hohenester and Trügler 20. A numerical model of a bowtie was constructed from two prisms with equilateral triangular bases of 80 nm side length, 30 nm heights, radii of curvature at the vertices of 7 nm and a gap size of 17 nm. The complex refractive index of silver from Johnson and Christy^27^ was used and a refractive index of 1.33 was assumed for the ambient medium. The bowtie was excited by a plane wave at normal incidence and polarized along the longitudinal direction and the plasmon scattering spectra were calculated at the far field. From the spectrum of an unloaded cavity, we calculated *γ*_pl_=0.25 eV, which is smaller than the typical experimental linewidth. However, the cavity quality factor *Q*=*ω*_pl_/*γ*_pl_=7.3 was only slightly higher than the measured quality factor. Simulations of scattering spectra for a bowtie loaded with QDs were performed with dots modelled as spheres of 8 nm located at various positions within the gap. The complex dielectric function of these dots was approximated by a Lorentz model with a high-frequency dielectric constant 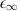
=6.1, *ω*_0_=1.8 eV, *γ*_0_=0.08 eV and an oscillator strength *f*=0.6 (refs 21, 22, 23). As the computed plasmon linewidth of the idealized bowtie structure was consistently smaller than the measured linewidth of a real bowtie, we scaled the linewidth of the QD accordingly.

The position-dependent coupling rate of a QD strongly coupled to the bowtie *g*(*r*) was evaluated using **E**(**r**), the electric field numerically calculated at the plasmon resonance energy *ω*_pl_=1.83 eV. To this end, the mode volume of the antenna was calculated using the approach of Koenderink^28^ and was used to normalize the energy density within the cavity, assuming it is occupied with a single photon of energy 1.83 eV. A map of *g*(*r*) plotted on a horizontal plane that intersects the bowtie at its half-height is shown in Fig. 3b. Supplementary Fig. 7 shows a similar calculation for a single prism (panel a), and compares the coupling rate of a bowtie and a single prism (panel b).

### Data availability

All relevant data are available from the authors on request.

## Additional information

**How to cite this article:** Santhosh, K. *et al.* Vacuum Rabi splitting in a plasmonic cavity at the single quantum emitter limit. *Nat. Commun.* 7:11823 doi: 10.1038/ncomms11823 (2016).

## Supplementary Material

Supplementary Information Supplementary Figures 1-8 and Supplementary Note 1.
