# Supplementary material for: Vacuum Rabi splitting in a plasmonic cavity at the single quantum emitter limit
Source: Nat Commun. 2016 Jun 13;7:ncomms11823. doi: 10.1038/ncomms11823 (PMC4909986; doi:10.1038/ncomms11823)
Supplement: Supplementary Information — Supplementary Figures 1-8 and Supplementary Note 1. [file ncomms11823-s1.pdf]

Supplementary Information

Supplementary Figure 1

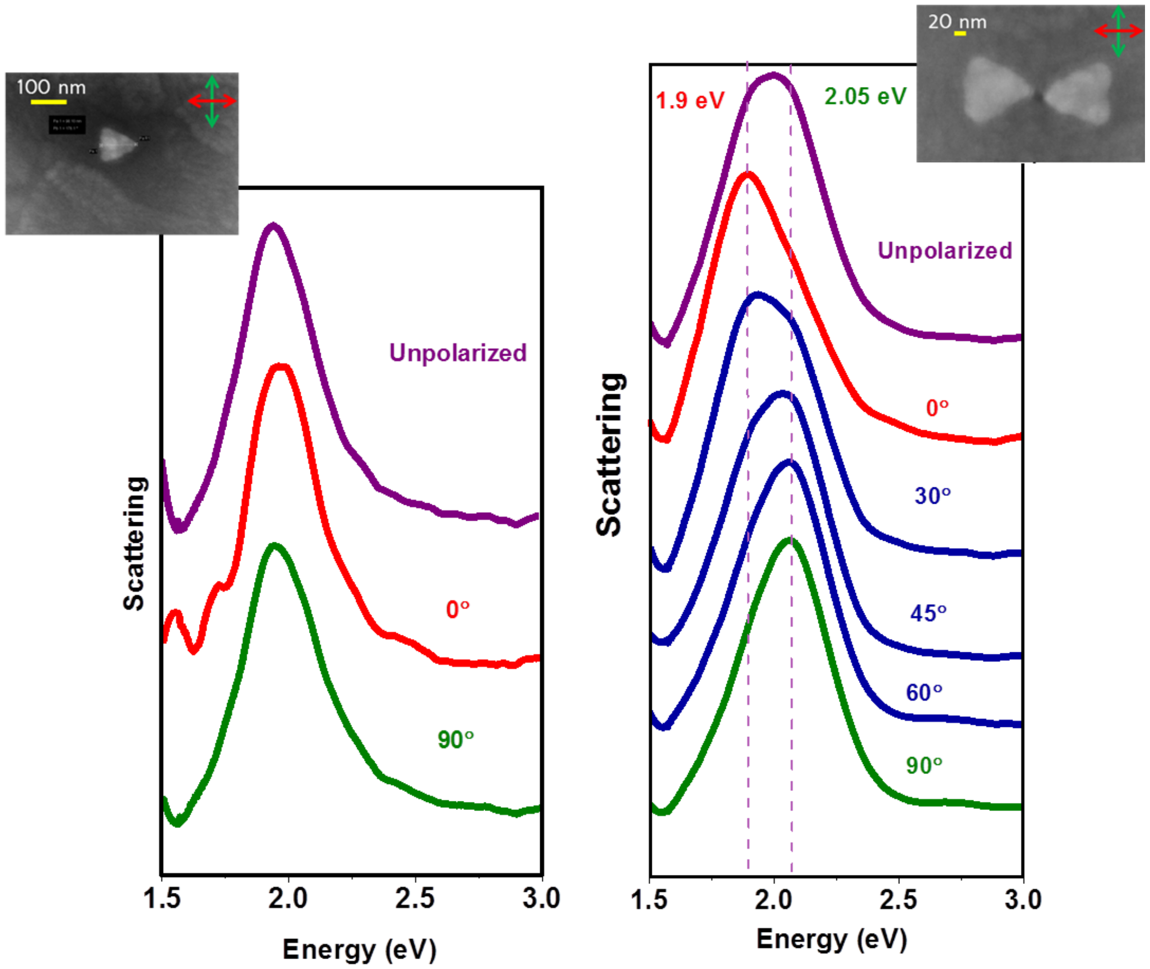

Dark field scattering spectra of an individual prism and an individual bowtie as a function of angle of polarization. The corresponding electron microscope images of the structures are also shown. Red and green arrows denote the direction of polarization parallel to the bowtie axis and perpendicular to it, respectively.

Supplementary Figure 2

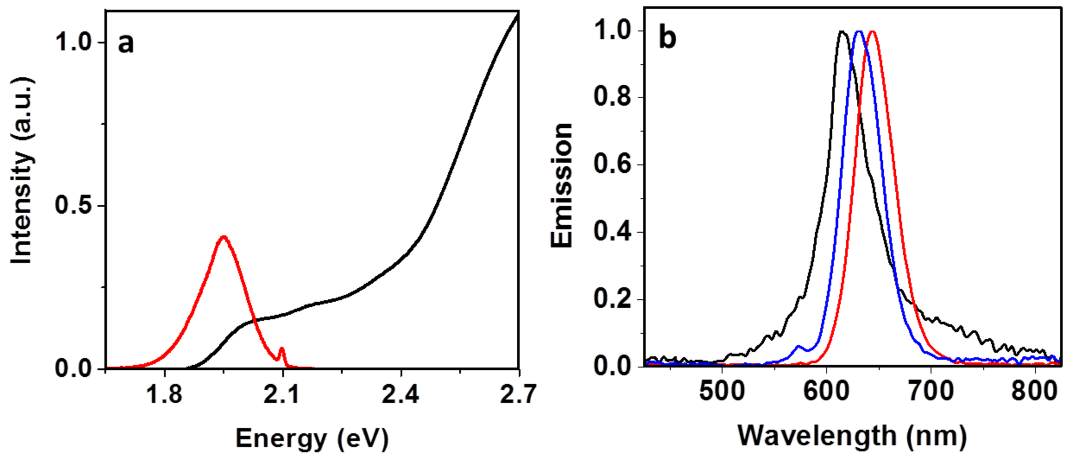

Spectroscopy of quantum dots in water. a. The figure shows absorption (black) and emission (red) spectra of CdSe/ZnS QDs in water. The lowest energy optical transition of the QDs (at 1.9 eV ≈ 660 nm) is at resonance with the longitudinal plasmon excitation seen in Supplementary Figure 1. **b.** Photoluminescence spectra of individual QDs deposited on a glass surface and excited with a 532 nm laser. Variations in the position of the luminescence peak are ascribed to heterogeneities in QD sizes. For a comment on the effect of the shape of the spectra of QDs on strong coupling see Supplementary Note.

Supplementary Figure 3

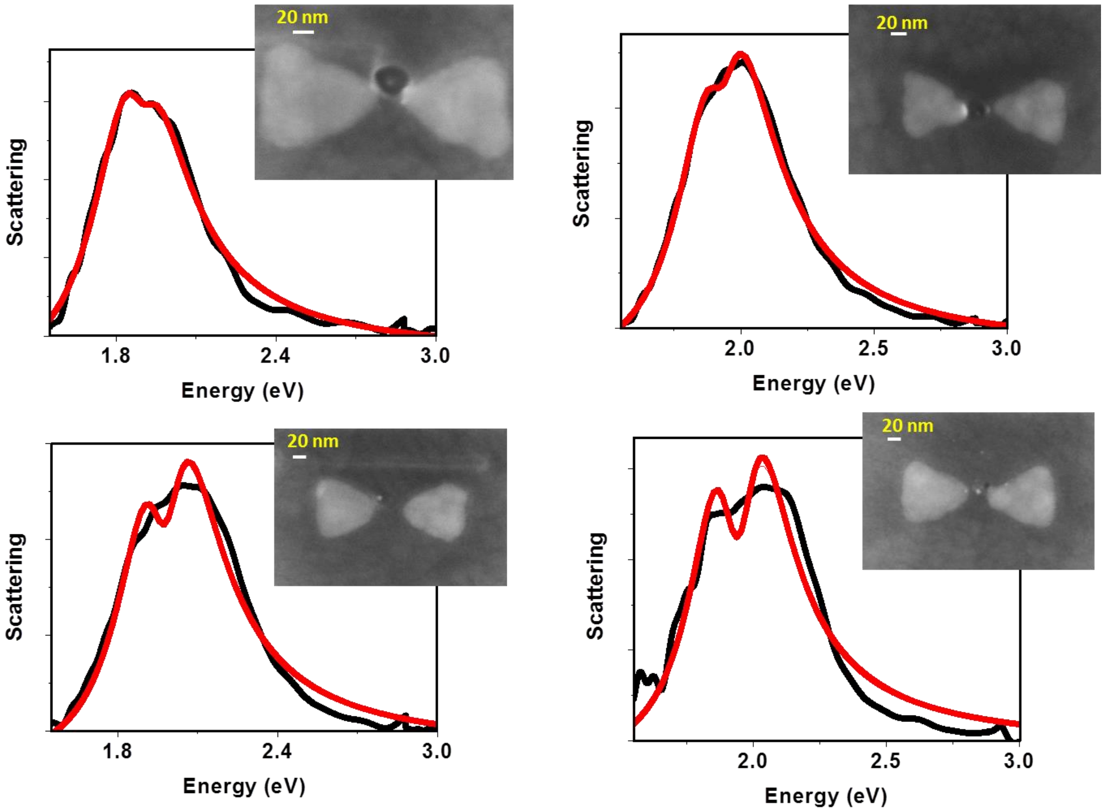

Scattering spectra and corresponding electron microscope images of bowties with a single QD in their gaps. Black lines are measured spectra, while red solid lines are fits to the coupled oscillator model. For these fits we fixed the value of the plasmon linewidth to the average value measured from empty bowties, 0.385 eV. The coupled oscillator model does not fit the two bottom spectra well, but the structure of the spectra and their widths clearly point to splitting (compare with Supplementary Figure 1).

Supplementary Figure 4

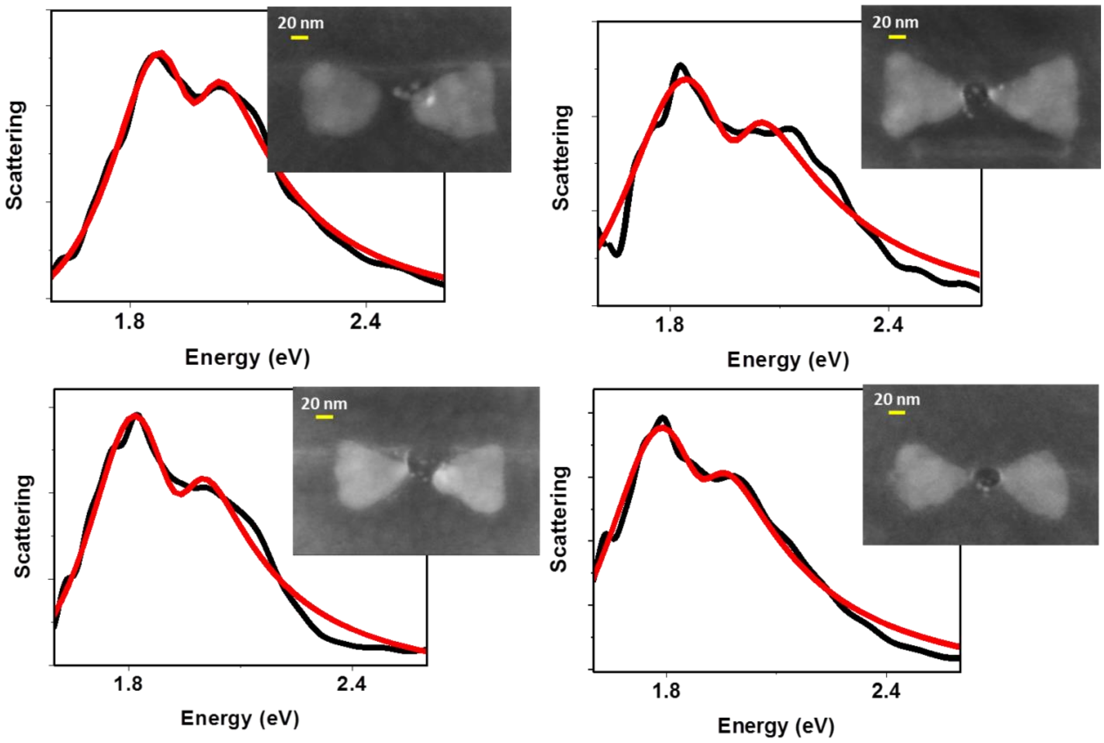

Scattering spectra of bowties with several QDs within the gap. The insets show scanning electron microscope images of the bowties. Black lines are measured spectra, while red solid lines are fits to the coupled oscillator model.

Supplementary Figure 5

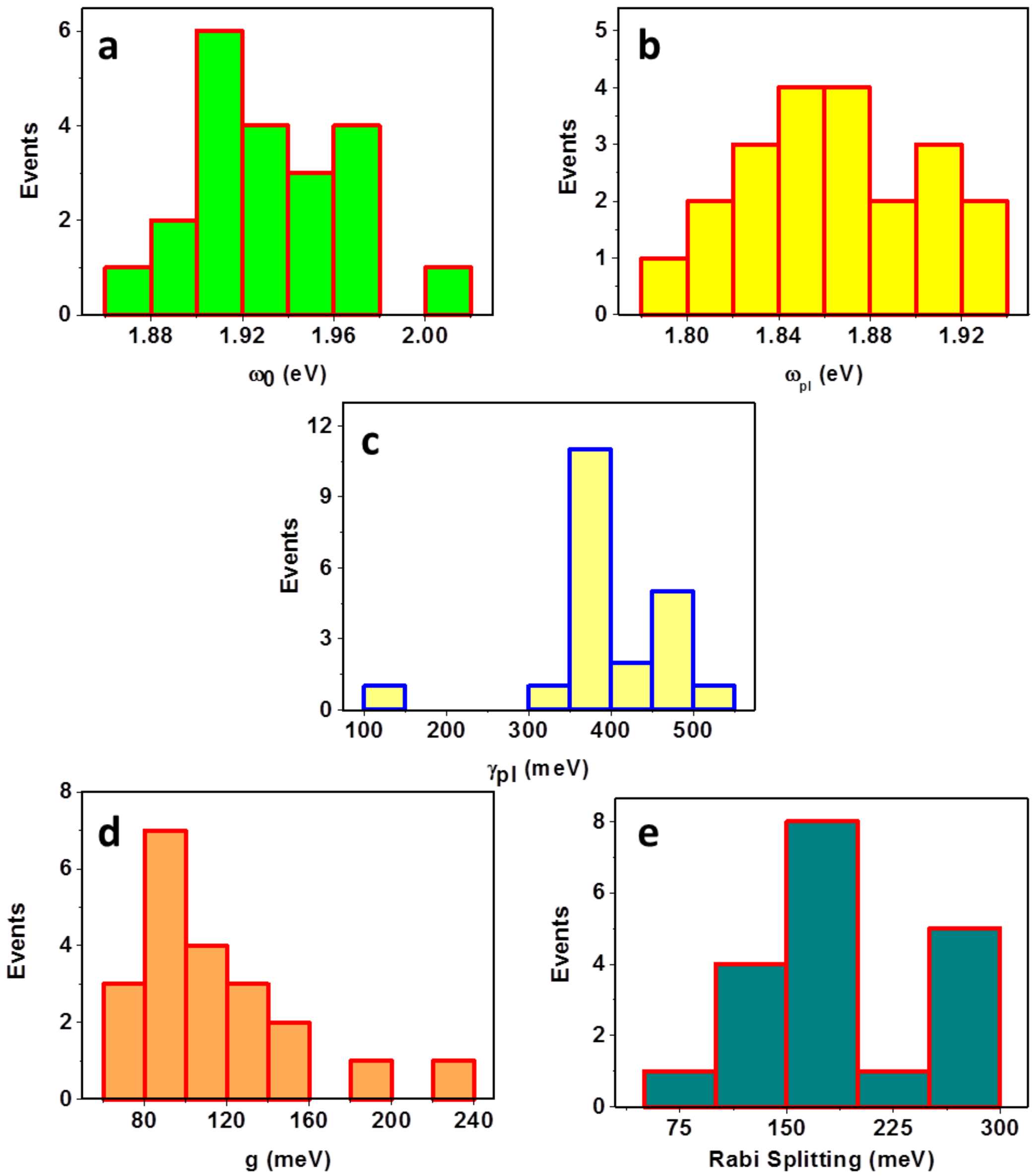

Parameters from coupled oscillator model fits to scattering spectra. The figure shows the fitting parameters obtained from fits of scattering spectra to the coupled oscillator model (equation 1 in Online Methods). **a.** Resonance frequency of the quantum emitter(s). **b.** Plasmon resonance frequencies. **c.** Plasmon linewidths. **d.** Coupling rates. **e.** Rabi splittings calculated directly from the fitted spectra.

Supplementary Figure 6

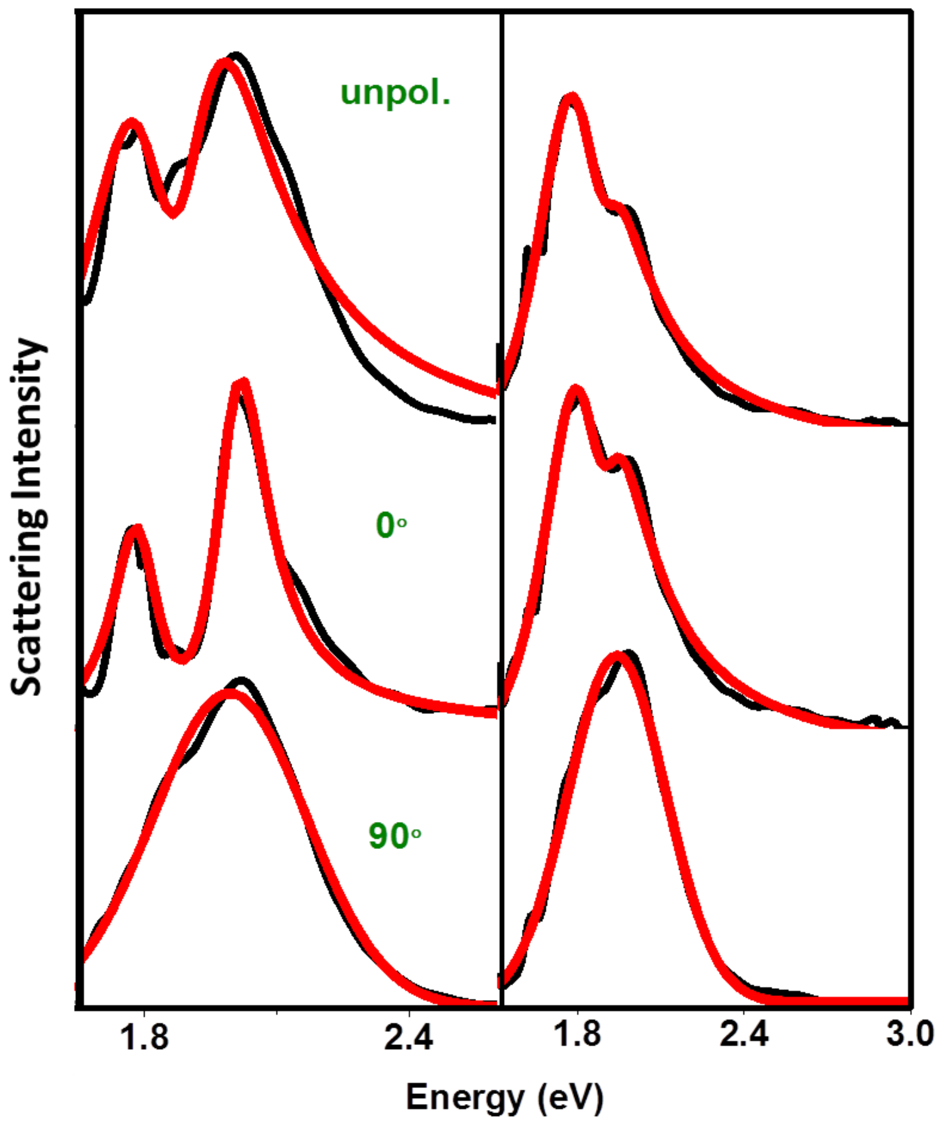

Polarization dependence of scattering spectra. Here two experiments similar to Fig. 2d of the main text are shown, with four QDs (left) and two QDs (right). Rabi splitting is observed even with unpolarized light (top row), but is strongest when the laser polarization is parallel to the bowtie long axis (0°, middle row). The splitting vanishes when the polarization is rotated by 90° (bottom row).

Supplementary Figure 7

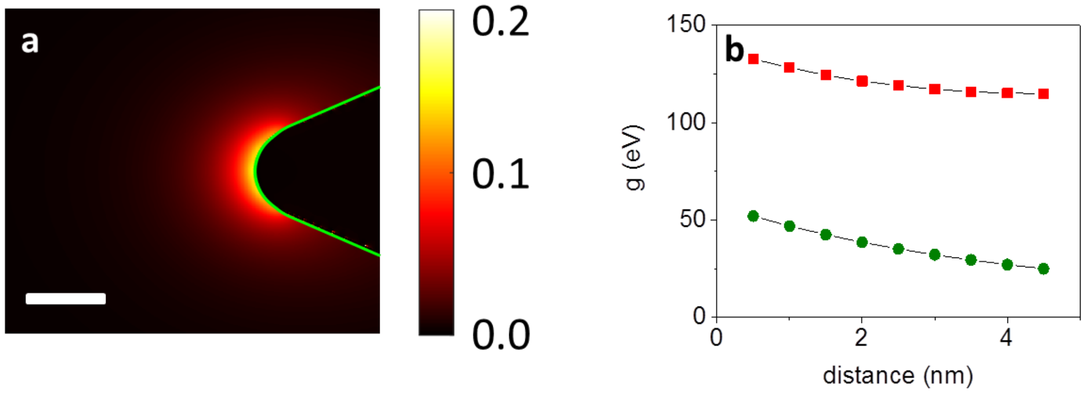

Electromagnetic simulations of coupling. a. The distribution of the coupling rate (in eV) of a quantum emitter with an oscillator strength of 0.6 near a single prism. The white bar represents 10 nm. **b.** Distribution of the coupling rate as a function of distance from one of the prisms of a bowtie (red) or from a single prism (green), along the center line and starting at the position where the edge of an 8 nm QD is 0.5 nm away from the prism.

Supplementary Figure 8

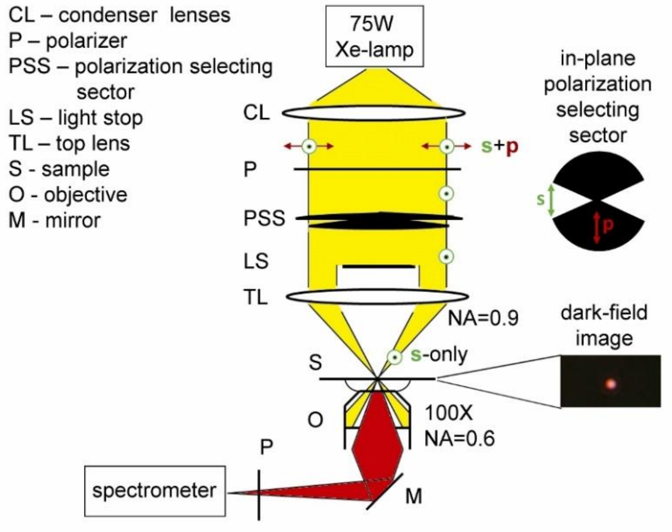

Schematic of the dark-field microspectromter. The setup was based on an inverted microscope equipped with a dark field condenser (NA=0.9), a 75 W Xenon lamp (Olympus) and a 100× oil immersion objective (NA=0.6). A combination of a light-stop with the top-lens of the condenser was used to exclude the light propagating along the normal to the sample plane, such that the light rays impinged on the sample at an angle of 70 degrees. A SpectraPro-150 spectrograph with a 1200 g/mm grating (Acton) and a Newton spectroscopy CCD camera (Andor Technology) were used to disperse the scattered light and register spectra. Polarization of the excitation light was controlled by a polarizer and an additional light-stop that passed the light only through a narrow selecting sector. When the sector was aligned such that the axis of the polarizer was normal to the bisecting line of the sector, the light at the sample was predominantly s-polarized.

Supplementary Note

The absorption spectrum of a semiconductor QD (Supplementary Figure 2) does not have a simple line shape like the emission spectrum, due to the absorption into higher energy states. How does that affect the coupling process? A good way to think about this problem is to envision this process in the time domain. First, a photon is injected into the cavity. As this photon bounces in the cavity it interacts with the quantum emitter. If the photon energy is in resonance with the emitter it absorbs the photon. Indeed, a QD contains more than a single exciton state. Each of the states that overlap the photon energy can absorb the photon. This photon has to be re-emitted. Typically a fast relaxation then brings the QD to the lowest-energy exciton, so the emission is from that exciton. However, irrespective of which exciton emits, unless the emission spectrum overlaps strongly with the cavity spectrum the photon will escape the cavity. If there is such overlap the photon stays in the cavity, and the process can be repeated more than one time, which is the essence of strong coupling.

The bottom line is that a prerequisite for strong coupling is good overlap between both the absorption and emission of the QD and the cavity. Whether one exciton state is involved or more should not matter, just as in the case of molecules the number of vibronic states involved does not seem to matter (a discussion of this issue for molecules is given in the review of Torma and Barnes, reference 9 of the manuscript, section 4.2). This issue might be approached through a detailed quantum calculation.
